# Publisher Correction: Costs of ribosomal RNA stabilization affect ribosome composition at maximum growth rate

**DOI:** 10.1038/s42003-024-06082-z

**Published:** 2024-04-11

**Authors:** Diana Széliová, Stefan Müller, Jürgen Zanghellini

**Affiliations:** 1 Department of Analytical Chemistry, University of Vienna, Vienna, 1090 Austria; 2 Faculty of Mathematics, University of Vienna, Vienna, 1090 Austria

**Keywords:** Computational models, Bioenergetics

Correction to: *Communications Biology* 10.1038/s42003-024-05815-4, published online 17 February 2024

In the PDF version of the manuscript, all instances where the constant k^0^ appears, it should read k^deg^ instead.

This has been corrected in PDF only.

