# Supplementary material for: Costs of ribosomal RNA stabilization affect ribosome composition at maximum growth rate
Source: Commun Biol. 2024 Feb 17;7:196. doi: 10.1038/s42003-024-05815-4 (PMC10874399; doi:10.1038/s42003-024-05815-4)
Supplement: Supplementary file 2 — Supplementary Information [file 42003_2024_5815_MOESM2_ESM.pdf]

# Costs of ribosomal RNA stabilization affect ribosome composition at maximum growth rate

**Diana Széliová[1,+], Stefan Müller[2,+], and Jürgen Zanghellini[1,+,*]**

[1]Department of Analytical Chemistry, University of Vienna, Vienna, 1090, Austria
[2]Faculty of Mathematics, University of Vienna, Vienna, 1090, Austria
[*]juergen.zanghellini@univie.ac.at
[+]All authors contributed equally to this work

## Supplementary information

## Contents

**1 Supplementary tables** 2
**2 Supplementary figures** 3
**References** 7

# 1 Supplementary tables

**Supplementary Table 1. Variables and model parameters**. Variables and model parameters for *E. coli* in different media, and for *Thermococcus*. If data for *Thermococcus* was not available, we used *E. coli* parameters from glucose minimal medium. LB, Luria-Bertani medium; Glc+AA, glucose + amino acids medium; Gly+AA, glycerol + amino acids medium; Glc, glucose minimal medium; Gly, glycerol minimal medium; Suc, succinate minimal medium. To formulate the constraints in Table 1 (main text), the kinetic parameters were converted from $s^{-1}$, $AA\,s^{-1}$, and $NT\,s^{-1}$ to $h^{-1}$, $AA\,h^{-1}$, and $NT\,h^{-1}$, respectively; molar masses were converted to $g\,mmol^{-1}$; in line with standard practice in constraint-based modeling, concentrations and fluxes are normalized to cell dry mass.

| Symbol | Name | LB | Glc+AA | Gly+AA | Glc | Gly | Suc | *Thermococcus* | Unit | Source |
|---|---|---|---|---|---|---|---|---|---|---|
| $n_{AA}$ | $\omega_{AA}/\omega_C$ | 0.61 | 0.61 | 1.18 | 0.61 | 1.18 | 0.92 | 0.61 | 1 | |
| $n_{NT}$ | $(\omega_{NT}-\omega_{AA})/\omega_C$ | 1.2 | 1.2 | 2.34 | 1.2 | 2.34 | 1.82 | 1.2 | 1 | |
| $n_{IC}$ | | 646 | | | | | | 646 | 1 | MC† CPLX-157 |
| $n_{EAA}, n_{ENT}$ | | 4875 | | | | | | 4875 | 1 | Estimate‡ |
| $n_{RNAP}$ | | 3498 | | | | | | 3338 | 1 | [1,2] |
| $n_{AF}$ | | 3900 | | | | | | 3900 | 1 | Estimate‡ |
| $n_{RNase}$ | | 813 | | | | | | 813 | 1 | MC† EG11259 |
| $\omega_C$ | Molar mass carbon source | 180 | 180 | 92 | 180 | 92 | 118 | 180 | $g\,mol^{-1}$ | |
| $\omega_{AA}$ | Molar mass amino acid | 109 | | | | | | 109 | $g\,mol^{-1}$ | BNID§ 104877 |
| $\omega_{NT}$ | Molar mass nucleotide | 324.3 | | | | | | 324.3 | $g\,mol^{-1}$ | BNID§ 104886 |
| $\omega_R$ | Molar mass ribosome | 2300000 | | | | | | 3040000 | $g\,mol^{-1}$ | [3,4] |
| $k^{cat}_{IC}$ | Carbon source import rate | 180 | | | | | | 180 | $s^{-1}$ | BNID§ 114686 |
| $k^{cat}_{EAA}$ | Enzyme turnover number | 10.5 | 8.5 | 7 | 5 | 3.5 | 2 | 5 | $s^{-1}$ | Estimate* |
| $k^{cat}_{ENT}$ | Enzyme turnover number | 10.5 | 8.5 | 7 | 5 | 3.5 | 2 | 5 | $s^{-1}$ | Estimate* |
| $k^{el}_{RNAP}$ | Transcription elongation rate | 85 | | | | | | 25 | $NT\,s^{-1}$ | [5,6] |
| $k^{el}_{R}$ | Translation elongation rate | 21 | | | | | | 8.3 | $AA\,s^{-1}$ | [5], ** |
| $k^{cat}_{AF}$ | Ribosome assembly rate | 1/120 | | | | | | 1/120 | $s^{-1}$ | BNID§ 102321 |
| $k^{deg}_{RNase}$ | RNase degradation rate | 88 | | | | | | 88 | $NT\,s^{-1}$ | [7] |
| $f^{act}_{RNAP}$ | RNAP activity | 0.31 | 0.242 | 0.188 | 0.15 | 0.144 | 0.132 | 0.15 | 1 | [3] |
| $f^{act}_{R}$ | Ribosome activity | 0.85 | | | | | | 0.85 | 1 | [8] |
| $\bar{k}^{el}_{RNAP}$ | Effective transcription elongation rate $\bar{k}^{el}_{RNAP}=f^{act}_{RNAP}k^{el}_{RNAP}$ | 26.35 | 20.57 | 15.98 | 12.75 | 12.24 | 11.22 | 12.75 | $NT\,s^{-1}$ | |
| $\bar{k}^{el}_{R}$ | Effective translation elongation rate $\bar{k}^{el}_{R}=f^{act}_{R}k^{el}_{R}$ | 17.85 | | | | | | 17.85 | $AA\,s^{-1}$ | |
| $K$ | Half-saturation constant | 0.2 | | | | | | 0.2 | 1 | |
| $\boldsymbol{c}$ | Species concentrations | | | | | | | | $mmol\,g^{-1}$ | |
| $\boldsymbol{v}$ | Metabolic fluxes | | | | | | | | $mmol\,g^{-1}\,h$ | |
| $\boldsymbol{w}$ | Protein synthesis fluxes | | | | | | | | $mmol\,g^{-1}\,h$ | |
| $\mu$ | Specific growth rate | | | | | | | | $h^{-1}$ | |
| $\phi^{R}_{i}$ | Ribosome allocation to protein $i$ | | | | | | | | 1 | |

† MetaCyc ID[9].
‡ estimated from an average protein length of 325 amino acids (BNID 108986) and an approximate number of proteins involved in amino acid/nucleotide synthesis (https://www.genome.jp/kegg/), or ribosome assembly[10].
§ BioNumbers ID[11].
* To consider the nutrient qualities of the different media, we assumed that $k^{cat}_{EAA}$ and $k^{cat}_{ENT}$ are proportional to the experimental growth rates (Suc: 0.4, Gly: 0.7, Glc:1, Gly+AA:1.4, Glc+AA: 1.7, LB: 2.1 $h^{-1}$). The growth rates were multiplied by 5 so that the maximum $k^{cat}_{EAA}$ corresponds to the average enzyme turnover number of 10 $h^{-1}$[12].
** An experimentally measured translation rate for *Thermococcus* is unavailable. However, archaeal transcription and translation are likely coordinated, similar to bacteria[13,14]. This suggests an upper bound for the translation rate at approximately $25/3 \approx 8.3\,AA\,s^{-1}$.

## 2 Supplementary figures

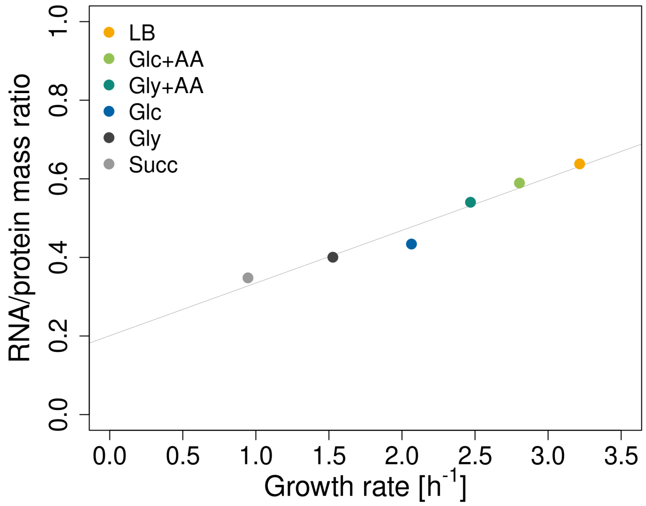


**Supplementary Figure 1.** Using variable translation elongation rate ($k_R^{el}$) leads to a predicted non-zero offset of RNA/protein ratio at zero growth rate. The points are predicted RNA/protein ratios for *E. coli* in six different conditions for the base model. The line is a linear fit. The used translation elongation rates ($k_R^{el}$) are: Succ, 12; Gly, 16.83; Glc, 21 ; Gly+AA, 20.17; Glc+AA, 21; LB, 22.25 $\mathrm{AA\,s^{-1}}$. The remaining parameters are listed in Supplementary Table 1.

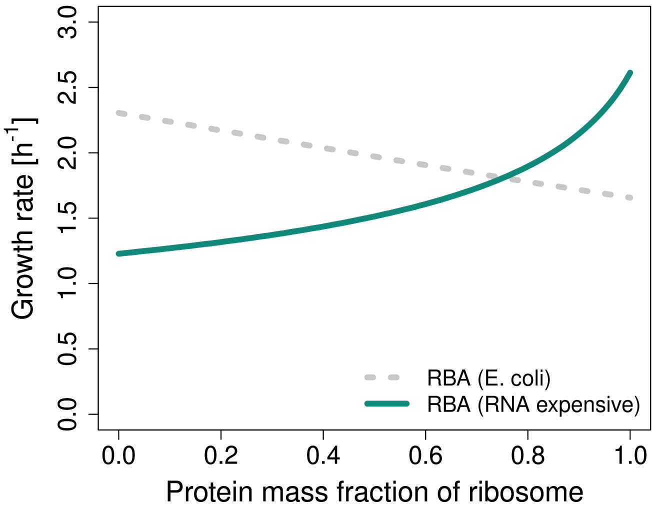


**Supplementary Figure 2.** Resource balance analysis (RBA) (base model) with realistic parameters for glucose minimal medium from Supplementary Table 1 (grey curve) vs. RBA with parameters that make ribonucleic acid (RNA) more expensive than proteins ($k_{RNAP}^{el} = 8.5\,\mathrm{NT\,s^{-1}}$, $k_R^{el} = 63\,\mathrm{AA\,s^{-1}}$, $n_{RNAP} = 52470$; green curve).

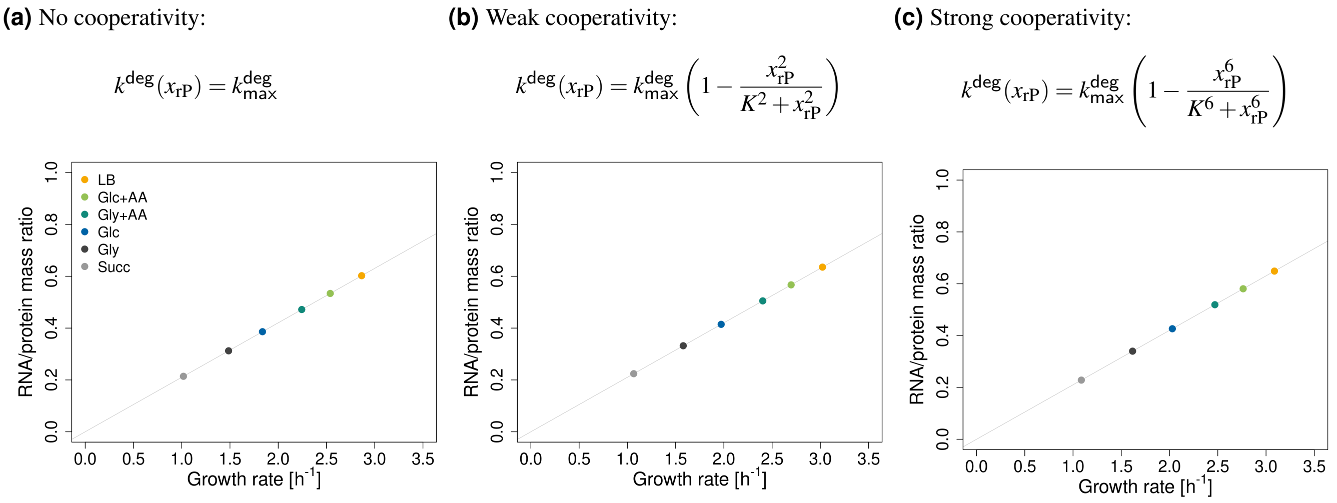


**Supplementary Figure 3.** Our extended model recapitulates linear relationship of RNA/protein mass ratio and growth rate for all three forms of ribosomal ribonucleic acid (rRNA) degradation function. The points are the predicted RNA/protein ratios for *E. coli* in six different conditions. The lines are linear fits.

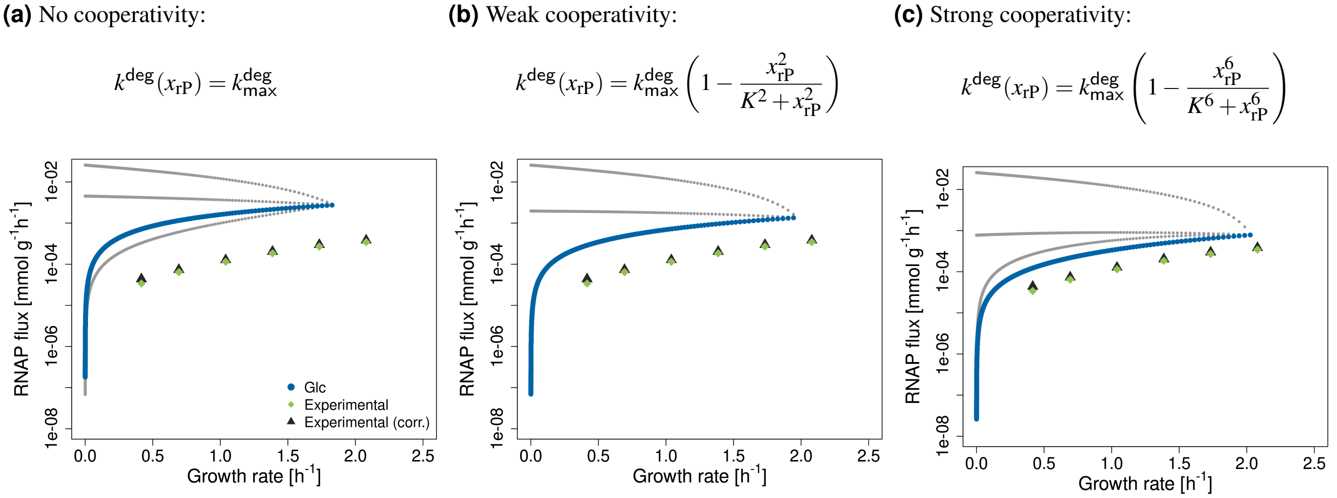


**Supplementary Figure 4.** RNA polymerase (RNAP) fluxes as functions of growth rate for the extended model for glucose minimal medium. Gray and blue lines are simulations. Light green diamonds are experimental data from Bremer and Dennis (2008)[8], black triangles are the data from Bremer and Dennis[8] corrected for rRNA degradation[15]. Data was converted to $\text{mmol}\,\text{g}^{-1}\,\text{h}^{-1}$ with *E. coli* dry masses from Bremer and Dennis[8] and the number of nucleotides in rRNA ($n_{\text{rRNA}}$) at $x_{\text{rP}} = 36\,\%$.

Unlike in the base model (Figure 2 b), we observe four instead of three alternative solutions (elementary growth vectors). The top gray trajectory represents solutions where RNase activity is higher than the enforced minimum given by Eqn. (5), which leads to increased RNAP flux. The middle gray trajectory are solutions where ribosomes accumulate in excess of what is needed for growth. This also increases rRNA degradation via equation (5) and therefore RNAP fluxes. Finally, the bottom gray solutions accumulate rRNA. Blue corresponds to elementary growth vectors where rRNA and ribosomes are not accumulating and rRNA is not degraded in excess, that is, constraints rRNA, cap R and min deg in Table 1 (main text) are fulfilled with equality.

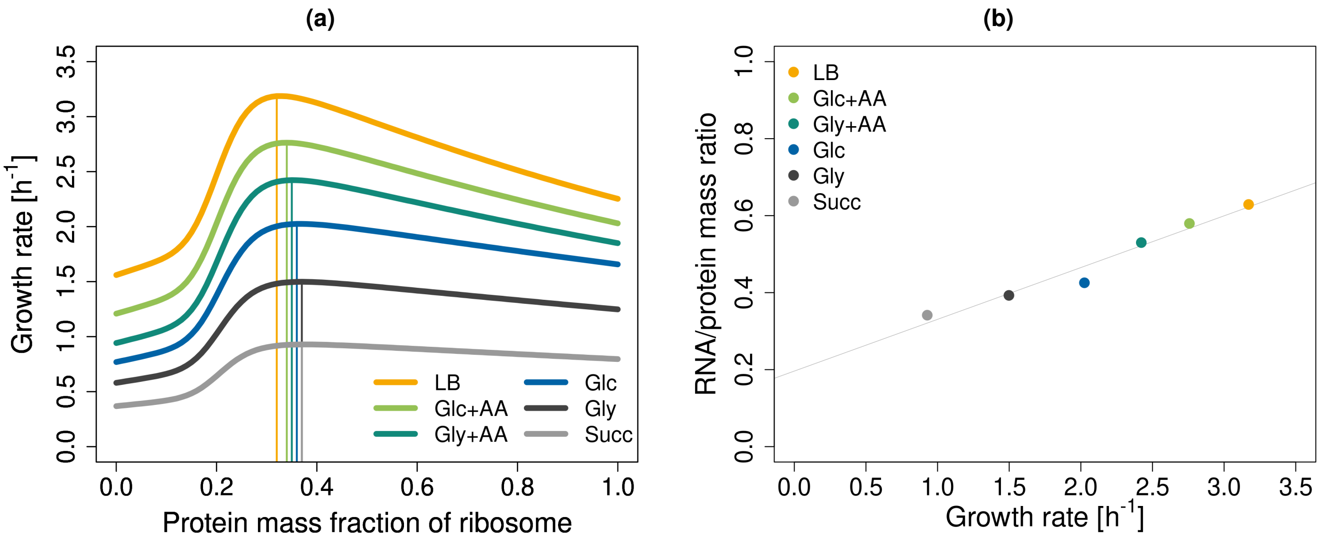


**Supplementary Figure 5.** Variable translation elongation rate has no impact on the predicted optimal ribosome composition, but it leads to a non-zero offset of RNA/protein ratio at zero growth rate (extended model). The translation elongation rates ($k_{\mathrm{R}}^{\mathrm{el}}$) are: Succ, 12; Gly, 16.83; Glc, 21 ; Gly+AA, 20.17; Glc+AA, 21; LB, 22.25 AA $s^{-1}$. The remaining parameters are listed in Supplementary Table 1. **(a)** Maximum growth rate for *E. coli* in six different conditions. **(b)** The points are the predicted RNA/protein ratios in six different conditions. The line is a linear fit.

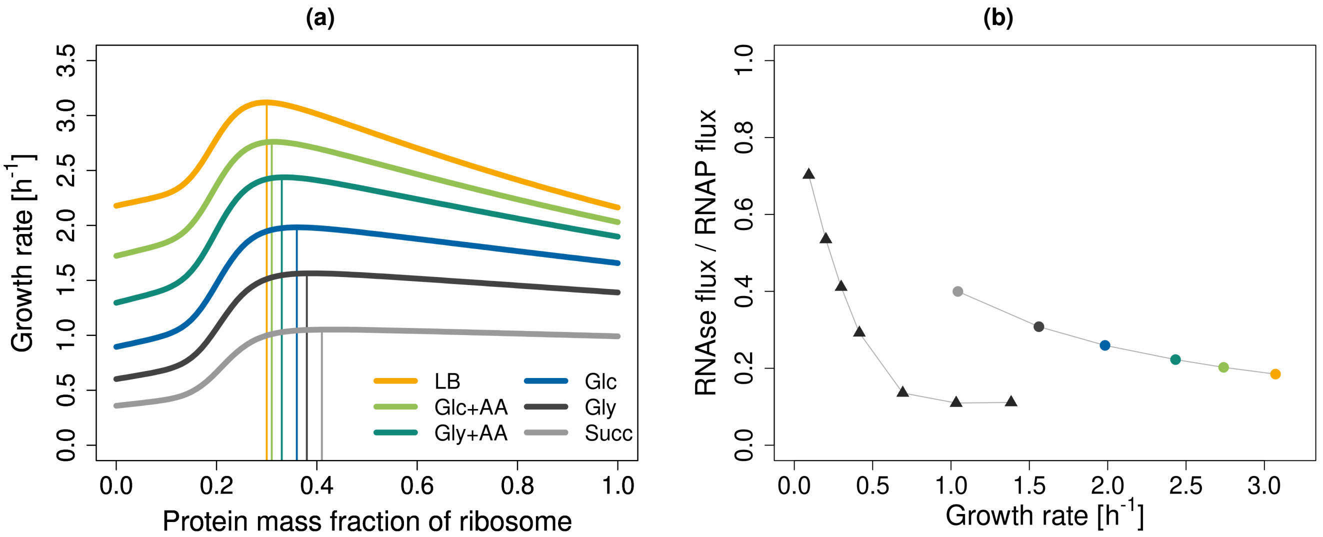


**Supplementary Figure 6.** Accounting for RNAP allocation improves predictions of RNase fluxes (extended model). RNAP allocations were included in the model by using the effective transcription rate $\bar{k}_{\mathrm{RNAP}}^{\mathrm{el}} = k_{\mathrm{RNAP}}^{\mathrm{el}} f_{\mathrm{RNAP}}^{\mathrm{act}} \phi_{\mathrm{rRNA}}^{\mathrm{RNAP}}$. The values for $\phi_{\mathrm{rRNA}}^{\mathrm{RNAP}}$ are: Succ, 0.18; Gly, 0.28; Glc, 0.42; Gly+AA; 0.52, Glc+AA, 0.6; LB, 0.65. The values of the other parameters are in Supplementary Table 1. **(a)** Maximum growth rate for *E. coli* in six different conditions. **(b)** Ratio of RNA degradation to RNA transcription (RNAse flux/RNAP flux) The circles are the predicted ratios of RNAse fluxes to RNAP fluxes at different conditions. The triangles represent experimental data from Gausing (1977)[15] (extracted from the original plot with WebPlotDigitizer[16]).

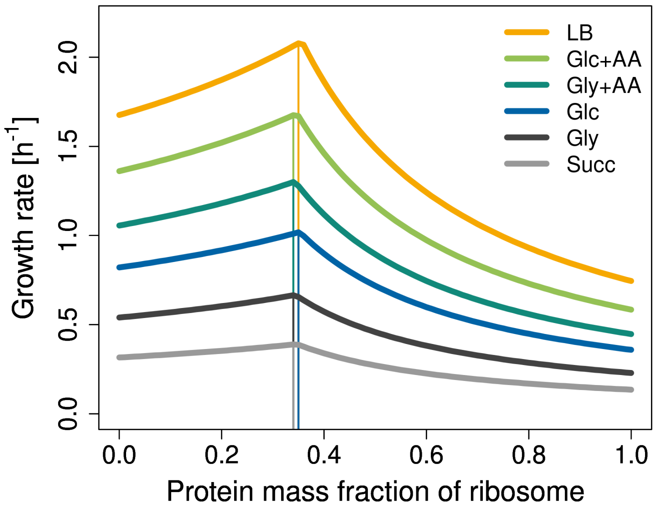


**Supplementary Figure 7.** The base RBA model with fixed ribosome allocations and parameters from Kostinski and Reuveni (2020)[3] in multiple growth conditions. For the definition of the ribosome allocations $\phi_{\mathrm{rP}}^{\mathrm{R}}$ and $\phi_{\mathrm{RNAP}}^{\mathrm{R}}$ see Eqn. (3). For the parameter values $(k_{\mathrm{R}}^{\mathrm{el}}, f_{\mathrm{R}}^{\mathrm{act}}, \phi_{\mathrm{rP}}^{\mathrm{R}})$ and $(k_{\mathrm{RNAP}}^{\mathrm{el}}, f_{\mathrm{RNAP}}^{\mathrm{act}}, \phi_{\mathrm{RNAP}}^{\mathrm{R}}, \phi_{\mathrm{rRNA}}^{\mathrm{RNAP}})$, see the original paper.

## References


1. Sutherland, C. & Murakami, K. S. An introduction to the structure and function of the catalytic core enzyme of Escherichia coli RNA polymerase. *EcoSal Plus* **8**, DOI: 10.1128/ecosalplus.ESP-0004-2018 (2018).
2. Jun, S.-H. *et al.* Direct binding of TFE$\alpha$ opens DNA binding cleft of RNA polymerase. *Nat. communications* **11**, 1–12, DOI: 10.1038/s41467-020-19998-x (2020).
3. Kostinski, S. & Reuveni, S. Ribosome composition maximizes cellular growth rates in E. coli. *Phys. Rev. Lett.* **125**, 028103, DOI: 10.1103/PhysRevLett.125.028103 (2020).
4. Acca, M. *et al.* Updating mass and composition of archaeal and bacterial ribosomes. Archaeal-like features of ribosomes from the deep-branching bacterium Aquifex pyrophilus. *Syst. Appl. Microbiol.* **16**, 629–637, DOI: 10.1016/S0723-2020(11)80334-6 (1993).
5. Bremer, H. & Dennis, P. Modulation of chemical composition and other parameters of the cell by growth rate. Escherichia coli and Salmonella: cellular and molecular biology. *Am. Soc. for Microbiol.* 1553–1568 (1996).
6. Gehring, A. M. & Santangelo, T. J. Archaeal RNA polymerase arrests transcription at DNA lesions. *Transcription* **8**, 288–296, DOI: 10.1080/21541264.2017.1324941 (2017).
7. Fazal, F. M., Koslover, D. J., Luisi, B. F. & Block, S. M. Direct observation of processive exoribonuclease motion using optical tweezers. *Proc. Natl. Acad. Sci.* **112**, 15101–15106, DOI: 10.1073/pnas.1514028112 (2015).
8. Bremer, H. & Dennis, P. P. Modulation of chemical composition and other parameters of the cell at different exponential growth rates. *EcoSal Plus* **3**, 10–1128, DOI: 10.1128/ecosal.5.2.3 (2008).
9. Caspi, R. *et al.* The MetaCyc database of metabolic pathways and enzymes. *Nucleic acids research* **46**, D633–D639, DOI: 10.1093/nar/gkz862 (2018).
10. Choi, E., Jeon, H., Oh, J.-I. & Hwang, J. Overexpressed L20 rescues 50S ribosomal subunit assembly defects of bipA-deletion in Escherichia coli. *Front. microbiology* **10**, 2982, DOI: 10.3389/fmicb.2019.02982 (2020).
11. Milo, R., Jorgensen, P., Moran, U., Weber, G. & Springer, M. BioNumbers—the database of key numbers in molecular and cell biology. *Nucleic Acids Res.* **38**, D750–D753, DOI: 10.1093/nar/gkp889 (2009).
12. Bar-Even, A. *et al.* The moderately efficient enzyme: Evolutionary and physicochemical trends shaping enzyme parameters. *Biochemistry* **50**, 4402–4410, DOI: 10.1021/bi2002289 (2011).
13. French, S. L., Santangelo, T. J., Beyer, A. L. & Reeve, J. N. Transcription and translation are coupled in Archaea. *Mol. Biol. Evol.* **24**, 893–895, DOI: 10.1093/molbev/msm007 (2007).
14. Proshkin, S., Rahmouni, A. R., Mironov, A. & Nudler, E. Cooperation between translating ribosomes and RNA polymerase in transcription elongation. *Science* **328**, 504–508, DOI: 10.1126/science.1184939 (2010).
15. Gausing, K. Regulation of ribosome production in Escherichia coli: synthesis and stability of ribosomal RNA and of ribosomal protein messenger RNA at different growth rates. *J. molecular biology* **115**, 335–354, DOI: 10.1016/0022-2836(77)90158-9 (1977).
16. Rohatgi, A. Webplotdigitizer: Version 4.6. https://automeris.io/WebPlotDigitizer (2022). Last accessed on 2023-6-7.
